# Hand-Focused Strength and Proprioceptive Training for Improving Grip Strength and Manual Dexterity in Healthy Adults: A Systematic Review and Meta-Analysis

**DOI:** 10.3390/jcm14196882

**Published:** 2025-09-28

**Authors:** Anna Akbaş

**Affiliations:** Institute of Sport Sciences, Department of Human Motor Behavior, Academy of Physical Education, 40-065 Katowice, Poland; a.akbas@awf.katowice.pl; Tel.: +48-322075168

**Keywords:** hand function, grip strength, pinch strength, manual dexterity, proprioceptive training, strength training, randomized controlled trials, healthy adults

## Abstract

**Background**: Hand function is critical for daily living, occupational performance, and sports. Optimal training approaches for healthy adults remain unclear. **Objective**: To evaluate the effects of hand-focused strength and proprioceptive training on grip strength, pinch strength, manual dexterity, maximum voluntary contraction, joint position sense, and force sense in healthy younger and older adults. **Methods**: PubMed, Google Scholar, Semantic Scholar, Web of Science, Cochrane CENTRAL and registers were searched until July 2025 for randomized controlled trials (RCTs) involving adults (≥18 years) without upper limb dysfunction. Studies with clinical populations, single-session interventions, or lacking pre–post outcome data were excluded. Risk of bias was assessed using the Cochrane RoB 2 tool. Random-effects meta-analyses (Hedges’ g) pooled pre–post change score differences for each outcome; subgroup analyses examined age, training type, and comparator. **Results**: Twenty-two RCTs (n = 1017 participants; 19–78 years) met the inclusion criteria. Strength and proprioceptive training produced a small-to-moderate improvement in grip strength (g = 0.44, 95% CI [0.23–0.64], 95%PI [–0.09, 0.96]) and a large improvement in manual dexterity (g = 1.11, 95% CI [0.52–1.71], 95%PI [–0.01, 2.23]). Effects on pinch strength were positive but non-significant (g = 0.63, 95% CI [–0.09–1.35], 95%PI [–1.38, 2.63]) and showed substantial heterogeneity. Moderator analyses indicated greater effects in older adults (g = 0.97) compared to younger adults (g = 0.18). Strength-only protocols showed significant effects, while combined protocols yielded smaller, non-significant effects; however, the difference between them was not statistically significant. Effects were also larger when compared with passive controls than with untrained hands. **Limitations**: Evidence quality was limited by high risk of bias, measurement variability, and small study numbers for some outcomes. **Conclusions**: Strength-focused hand training, particularly in older adults, yields meaningful improvements in grip strength and dexterity. Further high-quality RCTs are needed for under-studied outcomes.

## 1. Introduction

Hand function is a decisive factor in both elite performance and healthy aging. Optimal upper limb function underpins a wide range of daily [1], occupational [2], and athletic activities [3]. For athletes in sports requiring precise and sustained hand control—such as fencing or rock climbing—hand strength, fine motor coordination, and sensorimotor acuity are critical determinants of performance [4,5,6]. In older adults, the preservation of grip strength, dexterity, and proprioceptive function is strongly associated with independence, reduced fall risk, and improved quality of life [7,8,9].

Two broad training approaches are commonly used to enhance hand function: strength training, which targets force production, endurance, and neuromuscular efficiency [10], and proprioceptive training, which focuses on improving joint position sense, force modulation, and motor coordination [11,12,13]. While these methods are well established in rehabilitation, their application in healthy populations—both younger adults aiming to optimize performance and older adults seeking to maintain functional ability—remains less systematically understood.

Strength and proprioceptive training target different but complementary aspects of hand motor performance. Strength-focused programs increase the efficiency of grip and pinch actions by enhancing force production and endurance, while proprioceptive training sharpens sensory feedback from muscles, tendons, and joint receptors, thereby improving position sense, steadiness, and fine finger control [14,15]. Examples of proprioceptive methods include joint position matching, perturbation-based grip tasks, or haptic and vibrotactile feedback, each of which challenges the neuromuscular system in ways that go beyond pure resistance exercise. Strength protocols, when applied progressively, can also contribute to sensory improvements, influencing both intrinsic and extrinsic hand muscle control [16]. In summary, these two approaches offer the potential to reinforce both the muscular and sensory foundations of functional grip and skilled object manipulation. What remains uncertain, however, is whether these benefits—well documented in clinical and rehabilitation settings—translate into measurable improvements in healthy adults, which is the focus of the present review.

Several systematic reviews have examined the effects of resistance or proprioceptive training, but their scope has been limited. For example, Abe et al. [17] reviewed resistance exercise interventions for handgrip strength in healthy adults but found the evidence to be largely inconclusive due to small samples and lack of low-risk-of-bias RCTs, particularly in younger populations. Aman et al. [12] demonstrated that proprioceptive training can enhance sensorimotor function, but this synthesis was broad, covering multiple clinical groups and body regions rather than focusing on hand-specific outcomes in healthy adults. Similarly, Malwanage et al. [13] concentrated on proprioceptive training in Parkinson’s disease, providing insights for rehabilitation but not for prevention or performance contexts.

This leaves a critical gap: no systematic review has comprehensively examined hand-focused strength and proprioceptive training in healthy adults across the lifespan. Declining hand strength and proprioceptive acuity predict functional loss in older adults, while insufficient hand control can limit precision and power in athletes and workers. By integrating evidence from both younger and older healthy populations, the present review addresses the question: Does hand-focused strength and proprioceptive training improve grip strength, maximum voluntary contraction, joint position sense, force sense, and manual dexterity in adults without upper limb dysfunction?

## 2. Materials and Methods

### 2.1. Search Strategy and Study Identification

A comprehensive systematic literature search was finalized between 15 July and 31 July 2025, using the databases PubMed, Google Scholar, Semantic Scholar, Cochrane CENTRAL, Web of Science and registers (ClinicalTrails.gov and WHO ICTRP). The search covered all years of publication up to July 2025, with no lower date restriction; the earliest included study was published in 1999. The objective was to identify peer-reviewed randomized controlled trials (RCTs) investigating the effects of hand-focused strength and/or proprioceptive training on upper limb function in adult populations.

**Figure 1:**
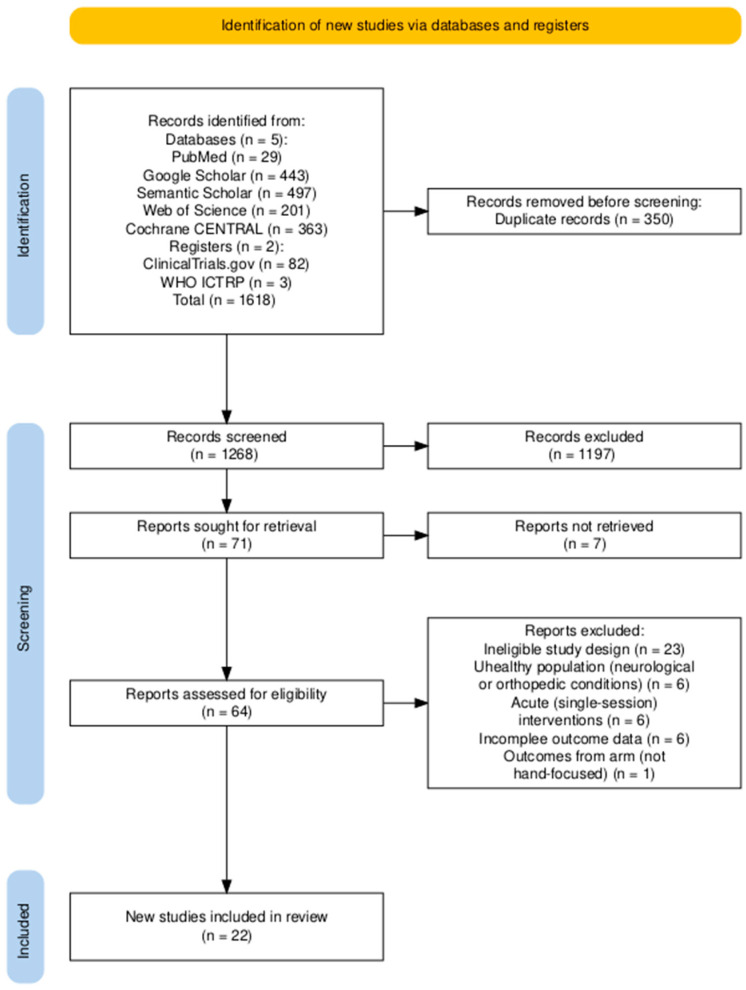
PRISMA 2020 flow diagram of study selection for the systematic review and meta-analysis. Diagram generated using the PRISMA2020 R package [18].

The search strategy was developed in accordance with the PRISMA 2020 guidelines (Preferred Reporting Items for Systematic Reviews and Meta-Analyses) (Figure 1), incorporating the 27-item checklist and flow diagram. Boolean operators and structured keyword combinations were used to ensure both sensitivity and specificity. The following search string was applied in databases and registers:

(“hand training” OR “hand exercise” OR “hand rehabilitation” OR “grip training” OR “pinch training”) AND (“proprioception” OR “proprioceptive training” OR “sensorimotor training” OR “joint position sense” OR “force sense”) AND (“grip strength” OR “maximum voluntary contraction” OR “MVC” OR “pinch strength”) AND (“dexterity” OR “manual dexterity” OR “fine motor skills” OR “hand function”) AND (“young adults” OR “older adults” OR “healthy adults” OR “non-disabled” OR “without upper limb dysfunction”) AND (“randomized controlled trial” OR “RCT” OR “clinical trial”).

In practice, this string was treated as a pool of keywords and subject headings; subsets of these terms were combined depending on the database interface to maximize sensitivity, since a single composite query did not always retrieve records.

Searches in Semantic Scholar were conducted via the Elicit interface, which supports natural-language queries structured according to the PICOS framework. The following query was used: “Does hand-focused strength and proprioceptive training improve grip strength, maximum voluntary contraction, joint position sense, force sense, and dexterity in young and older adults without upper limb dysfunction?”

The search protocol was guided by the PICOS framework, which defined both search logic and eligibility criteria as follows:

- P (Population): Adults (≥18 years) without diagnosed upper limb musculoskeletal or neurological conditions and with no upper limb surgery within the previous 6 months.
- I (Intervention): Structured hand-focused strength training, proprioceptive exercises, or combined protocols.
- C (Comparator): Passive control, no intervention, conventional physiotherapy, or alternative active exercise.
- (Outcomes): At least one of the following: grip strength, maximum voluntary contraction (MVC), pinch strength, joint position sense (JPS), force modulation, or manual dexterity.
- S (Study Design): Only randomized controlled trials with a control group and pre- and post-intervention assessments conducted over more than one session were included.

As this review was based entirely on previously published studies, ethical approval was not required. This review was not registered in a prospective register, and no formal review protocol was prepared. However, outcomes and subgroup analyses were pre-specified, while the handling of multiple arms or hands was refined according to established methodological guidance and applied consistently.

### 2.2. Inclusion and Exclusion Criteria

Studies were eligible for inclusion if they met all of the following criteria:

(1) Study Design: The study employed a randomized controlled trial design with at least two comparison groups. These could include a separate control group (passive or active), an internal control (e.g., untrained limb), or two or more distinct intervention arms.
(2) Intervention Duration: The intervention extended beyond a single session and evaluated longitudinal effects rather than acute responses.
(3) Intervention Type: The intervention included structured hand strength training, proprioceptive training exercises, or a combination of both.
(4) Measurement Protocol: The study included both pre- and post-intervention assessments using validated measurement tools.
(5) Outcome Measures: At least one of the following outcomes was assessed quantitatively: grip strength, MVC, JPS, force sense or force modulation, or manual dexterity.
(6) Participant Age: All participants were aged 18 years or older at the time of enrollment.
(7) Participant Health Status: Participants were free from any diagnosed upper limb musculoskeletal conditions, neurological disorders affecting upper limb function, or recent upper limb surgery within the previous six months. Participants may include individuals with stable age-related functional decline or specific chronic conditions (e.g., hypertension, arthritis, dynapenia, or self-perceived hand function decline) provided these conditions are not acutely decompensated and do not severely impair upper limb movement or cognitive function

Studies were excluded if they met any of the following criteria:

(1) The study did not employ a randomized controlled trial design with at least two comparison groups. This includes single-group pre-post studies, cross-sectional designs, feasibility or pilot studies without a control (external or internal) or multiple intervention arms (e.g., [19]), as well as quasi-experimental studies without randomization.
(2) The intervention was limited to a single session or assessed only acute effects.
(3) The intervention did not include hand strength or proprioceptive training (e.g., [20]).
(4) Pre- and post-intervention measurements were not reported.
(5) None of the eligible outcomes were assessed.
(6) The study included participants under 18 years of age or individuals with neurological or orthopedic upper limb conditions, or those who had undergone upper limb surgery within the past six months (e.g., [21]).
(7) The study did not report a complete set of numerical data (i.e., pre- and post-intervention means and standard deviations). Studies were excluded if values were non-numerical or if effect sizes had to be estimated indirectly (e.g., [22,23]).

Studies were grouped for each synthesis according to the type of intervention (strength training, proprioceptive training, combined strength–proprioceptive training), participant age group (younger adults, 18–35 years; older adults, ≥60 years), and type of comparator (passive control, active control, untrained hand). Intervention characteristics and outcome measures were tabulated and compared against the pre-specified subgroups defined in the protocol to determine eligibility for each synthesis.

### 2.3. Outcomes

The primary outcomes sought were as follows:

- Grip strength—maximum voluntary isometric force produced by the hand reported in kilograms or Newtons.
- Pinch strength—maximum isometric force generated between the thumb and index finger, typically measured with a standardized pinch gauge.
- Maximum voluntary contraction—peak voluntary force effort assessed through surface electromyography (EMG) of specific hand muscle groups during sustained maximal contraction.
- Joint position sense—accuracy of reproducing specific joint angles at the wrist or fingers, expressed as absolute or variable error in degrees.
- Force sense—accuracy or steadiness of producing target submaximal forces, reported as force error or coefficient of variation.
- Manual dexterity—performance on standardized timed tests simulating fine or gross motor tasks (e.g., Nine-Hole Peg Test, Purdue Pegboard Test, Jebsen–Taylor Hand Function Test, Box and Block Test).

For each included study, all available post-intervention results compatible with these outcome domains were sought, regardless of the specific measurement tool, provided it was validated for the target construct. Where multiple compatible measures or time points were reported for the same outcome, the following decision rules were applied:

- When both hands were tested, data from the trained/dominant and non-dominant hand were extracted and included in the primary analysis. For bilateral designs where the contralateral hand served as comparator, the Cochrane unit-of-analysis correction was applied by halving the effective sample size (N/2).
- When multiple post-intervention assessments were reported, the first time point after the completion of the intervention was used.

### 2.4. Study Selection Process

Two reviewers (A.A. and a research assistant) independently screened the titles, abstracts, and full-text articles retrieved through the search to determine eligibility based on the predefined inclusion and exclusion criteria (Figure 1). Discrepancies were resolved through discussion until consensus was reached. In addition to database searches, Semantic Scholar was queried via the Elicit interface, which employs machine learning algorithms to identify and prioritize potentially relevant studies, thereby supporting the screening process.

### 2.5. Data Collection Process

Two reviewers (A.A. and a research assistant) independently extracted data from each included study using a pre-defined extraction form. Extracted variables included study design, participant characteristics (sample size, age, sex), intervention and comparator details (type, duration, frequency), outcome measures, and pre- and post-intervention means and standard deviations for all relevant outcomes. The process was supported by the NotebookLM AI platform (Google LLC, latest release as of August 2025), which was used to locate and extract numerical data from PDF study reports. All automatically extracted values were manually verified against the original full-text articles. Any discrepancies in the extracted data were resolved by discussion until consensus was reached. No study authors were contacted, as all required data were available in the published reports.

### 2.6. Risk of Bias Assessment

Risk of bias was assessed for each included randomized controlled trial using the original Cochrane Risk of Bias 2 (RoB 2) Excel tool, which applies automated algorithms to generate domain-level and overall judgments based on reviewer responses. Two reviewers (A.A. and a research assistant) independently evaluated each study. Discrepancies in responses or judgments were resolved through discussion until consensus was reached.

### 2.7. Statistical Methodology for Meta-Analysis

For each study and all the outcomes, pre–post differences (Δ) were calculated separately for experimental and control groups, and these deltas were used as the basis for effect size calculations. All outcomes were coded so that higher standardized mean differences reflected better performance. For time-based dexterity tests (e.g., Nine-Hole Peg Test, Grooved Pegboard), values were inverted prior to analysis so that lower completion times corresponded to higher performance. All analyses were conducted using Jamovi v.2.6 (MAJOR module).

Meta-analyses were conducted using standardized mean differences (SMD; Hedges’ g) as the effect size. When the standard deviation of the change score (SDΔ) was not reported, it was calculated according to the Cochrane Handbook formula, assuming a pre–post correlation of r = 0.5. To assess robustness, sensitivity analyses were conducted with r = 0.3 and r = 0.7. A random-effects model was applied, with heterogeneity estimated using the restricted maximum likelihood (REML) method (Viechtbauer, 2005 [24]). Between-study variability was assessed using τ^2^, Cochran’s Q test (Cochran, 1954 [25]), and the I^2^ statistic. When heterogeneity was present (τ^2^ > 0), a 95% prediction interval for the true effects was also reported.

Influential studies and potential outliers were assessed using studentized residuals and Cook’s distances. Outliers were identified using Bonferroni-adjusted thresholds based on the standard normal distribution, and influence was defined as a Cook’s distance exceeding the median plus six times the interquartile range. Funnel plot asymmetry was evaluated using the rank correlation test and Egger’s regression test, with the standard error of effect sizes as the predictor. No formal assessment of the certainty or confidence in the body of evidence (e.g., GRADE) was conducted.

### 2.8. Statistical Methodology for Meta-Regression

Potential sources of heterogeneity were examined through subgroup and meta-regression analyses. Subgroups were predefined by participant age group (younger adults vs. older adults), intervention type (strength training, proprioceptive training, combined), and comparator type (passive control, active control, untrained hand). Differences between subgroups were evaluated using a mixed-effects model, with between-subgroup heterogeneity assessed via the Q statistic. In addition, meta-regressions were conducted to formally test whether these moderators explained between-study variability. Mixed-effects models were fitted using restricted maximum likelihood estimation, with inverse-variance weights proportional to the precision of each study’s effect size (1/SE^2^). All models were run in Jamovi v.2.6 (MAJOR module), and each moderator analysis included 19 study arms.

## 3. Results from the Data Search and Meta-Analysis

### 3.1. Participants

This systematic review included 1017 participants across all intervention and control groups (Table 1). Mean ages spanned from 19.70 ± 1.41 years [26] to 77.55 ± 4.40 years [27], with several studies including participants aged 18–25 years and covering younger adults (~18–30 years) (e.g., [1,6,26,28]) and older adults (≥60 years). Several trials explicitly targeted older populations (e.g., [7,8,27,29,30,31,32]).

Sex distribution varied widely: mixed-sex cohorts with female predominance (e.g., [1] Group A 64% female and Group B 50% female; [33] Group A 67% female and Group B 84% female; [34] 86.7% female in both groups); mixed-sex cohorts with male predominance (e.g., [31] Bimanual Digit group 60.9% male, Right-Hand group 68% male; [35] Control group 70% male); all-female samples (e.g., [7] both groups 100% female; [36] training group 100% female for the intervention groups); and all-male samples (e.g., [26] 0% female; [28] 0% female; [37] 0% female). Some studies did not report a detailed sex breakdown for all participants or groups (e.g., [6,38]).

Sample sizes ranged from 7 participants per group [39] to 169 total participants in the largest study [38]. Notably, no single experimental arm exceeded 45 participants. Across studies, total sample sizes were most commonly 20–60 participants, with individual intervention arms typically including 10–30 participants.

**Table 1:** Characteristics of included studies.

Study	Type	Groups	N	F%	Age (Years)	Intervention Description	Dur. (wks)	Freq.	Comparator	Outcomes	Main Result
Abbas et al. (2020) [1]	S	Strength Training	41	64	19.95 ± 1.62	Isometric handgrip (dominant hand): 1 min sets, 4 s max grip/2 s rest	6	2/wk	Active control; within subject—NDH as passive control	Grip strength, Pinch strength	Grip ↑ in both groups; vibration = no added effect
	C	Strength + vibration	36	50	20.05 ± 1.54	As above + 5 min UL vibration (30 Hz, 2 mm)					
Arshad et al. (2023) [8]	S	Resistance Exercise	12	58	69.67 ± 6.28	Handgrip RT (baseline)	4	3/wk	Active control	Grip strength	Grip and function ↑ in both; no difference in older adults
	C	Finger Exercise	12		72.50 ± 7.06	Handgrip RT + finger exercises (pinch, flick, crook, count, press)					
Bartolomé et al. (2021) [28]	S	Normothermia	29	0	21.75 ± 0.34	Handgrip: 10 × 10 reps, 45 s rest (normothermia)	3	2/wk	Active control; within subjects—NDH as passive control	Grip strength	Heat training ↑ strength bilaterally; no effect in normothermia
		Heat-exposed	25	0	21.23 ± 0.55	Same protocol in 100 °C dry sauna					
Bastone et al. (2020) [27]	S	Resistance Training	20	65	77.55 ± 4.40	Home-based supervised PR training (bands, dumbbells, ankle weights)	12–13	3/wk	Active and passive control	Grip strength	RT ↑ strength and function; nutrition ↑ gait only; combo = no added effect.
		Supplementation	20	74	76.50 ± 5.50	Protein supplement, 40 g/day.		1/day			
		Resistance Training plus Supplementation	20	83	76.95 ± 7.61	PR training + protein (40 g/day)		1/day			
		Control	20	78	72.50 ± 7.88	Usual daily routine (no intervention).					
Cuppone et al. (2015) [39]	P	HVTF—Haptic and Vibrotactile Feedback	7	79	27.92 ± 3.5	Robot-assisted wrist proprioception + haptic + vibrotactile feedback	3 days	1/day	Active control	Joint position sense	HVTF ↑ proprioception > HF
		HF—Haptic Feedback	7			Same, but haptic only (no vibrotactile)					
Gerodimos et al. (2021) [7]	S	Training	18	100	70.28 ± 4.01	Bilateral handgrip (10–15 min, 4–6 × 8–15 reps; balls, grippers)	8	2/wk	Passive control	Grip strength	Handgrip ↑ strength (9–10%) and endurance (14–27%); controls → no change
		Control	18	100	70.67 ± 3.22	No training					
Kumar & Nale (2023) [33]	P	Rhythmic Stabilization	18	67	44.56 ± 6.9	Isometric finger holds (dominant hand), 12 × 6 s/finger	4	3/wk	Active control	Grip strength, Pinch strength, Manual dexterity	Rhythmic stabilization ↑ all outcomes > control
	S	Combination of Isotonic Technique	19	84	45.26 ± 6.24	Single-finger concentric, isometric and eccentric contractions vs. manual resistance					
Laidlaw et al. (1999) [40]	S	HL—Heavy-load training)	8	50	68.3 ± 2.2	FDI training, heavy load (80% 1-RM)	4	3/wk	Active and passive control	Pinch strength, Force sense	Both ↑ strength; HL > LL for MVC
		LL—Light-load training	8	50	70.4 ± 2.0	FDI training, light load (10% 1-RM)					
		Control	16	69	72.4 ± 1.7 (SE)	No training					
Lee et al. (2024) [32]	S	Bi-RBT—Bimanual RBT without FES	11	73	64.54 ± 3.93	Bimanual resistance band (wrist + elbow flex/extend)	4	1/wk	Active control	Grip strength, Pinch strength, Force sense	Bi-RBT+FES ↑ unimanual force control in older adults
	C	Bi-RBT+FES—Bimanual RBT with FES	11		64.81 ± 2.82	Same + FES to UL muscles during movement					
Losana-Ferrer et al. (2018) [35]	C	MI—Motor Imagery	20	65	27.40 ± 11.20	2 × 10 × 3 s max isometric grip (tennis ball) + motor imagery	10 days	1/day	Active control	Grip strength	AO met ≥ 6 kg MCID; AO and MI ↑ strength and EMG > control
		AO—Action Observation	20	10	30.15 ± 13.24	Same + action observation (task video)					
		Control	20	30	33.30 ± 15.28	Daily: 10 max isometric grips					
Manca et al. (2016) [41]	S	Training	17	29	24.6 ± 5.4	Max-intensity unilateral isometric R-FDI (key pinch, 5 × 10 × 5 s) + visual/auditory feedback	4	3/wk	Passive control	Grip strength, Pinch strength	Training ↑ bilateral, task- and spatial-specific strength
		Control	17	35	26.3 ± 6.4	No training					
Marmon et al. (2011) [29]	C	Practice	15	52	74.9 ± 3.8	6 sessions Grooved Pegboard practice	6 sessions	2–4/wk	Passive control	Pinch Strength, Force sense, Manual Dexterity	Practice ↑ pegboard, steadiness and pinch; control → no change
		Control	8		75.0 ± 5.7	No training					
Mathews & Paul (2022) [30]	C	Experimental group	9	33	71.78 ± 5.97	Functional tasks (bottle carry, paper crumple, typing, buttoning)	4	3/wk	Passive control	Grip strength, Manual dexterity	Task-oriented training ↑ gross/fine dexterity and grip
		Control group	7	29	71.42 ± 5.38	Usual daily activities (no training)					
Naito et al. (2021) [31]	P	BM—Bimanual Digit Training (exp)	23	39	71.7 ± 4.3	Bimanual digit exercises (same/different finger actions)	8–9	1/day	Active and passive control	Manual dexterity	BM digit training > RH; ↓ ipsilateral motor cortex activity
		RH—Right-Hand Training	25	32	70.6 ± 4.2	Unimanual digit exercises (R-hand, matched tasks)					
		Younger Adults	31	29	22.1 ± 1.8	No training					
Pereira et al. (2011) [38]	P	Experimental	86	61	22.36 ± 1.60/41.60 ± 7.43	Unsupervised precision/dexterity: rice pickup, nuts/bolts, threading, coin flip	1	5	Passive control	Manual dexterity	Gross skill transfer retained 1 mo; fine dexterity transfer inconsistent (hand and age dependent)
		Control	83	55	23.24 ± 1.95/38.82 ± 7.81	No training					
Sarasso et al. (2018) [42]	P	Experimental group	20	50	22.61 ± 1.48	Somatosensory training (R-hand, blindfolded: textures, fabrics, point distances, shapes)	2	5	Active control	Manual dexterity	RH dexterity ↑ in both; only experimental ↑ all tests; somatosensory training → brain lateralization
	C	Control group	19	42	23.59 ± 2.20	Blindfolded ball manipulation (seated)					
Tan et al. (2024) [6]	S	Eccentric training	19	n/a	20.21 ± 0.25	Wrist flexor training (dom. hand): 3 × 6 × 5 s eccentric ext.	4	3	Active control; within subject—NDH as passive control	Grip strength	Grip ↑ bilaterally in both groups; eccentric = concentric
		Concentric training	20	n/a	20.05 ± 0.28	Concentric wrist flexion curls: 3 × 6 reps					
Taraf & Özal (2022) [26]	S	Exercise	27	0	19.70 ± 1.41	Dom. wrist flex/extend (dumbbell, 70% max, 4 sets to fatigue)	4	3	Passive control; within subject—NDH (effect of transfer)	Grip strength	Exercise ↑ grip bilaterally; control → no change
		Control	27	0	20.15 ± 1.66	No training					
Thomas et al. (2008) [36]	S	Training	9	100	24.6 ± 2.6	Home-based UB resistance (push-ups, dips, shoulder stab.): 3 × 10 (wk1–4), 3 × 15 (wk5+)	8	3	Passive control	Grip strength	UB resistance ↑ R-hand grip > control
		Control	11			No training					
Sakshi and Chaitali (2024) [34]	S	BFRT—Blood Flow Restriction Therapy	40	87	21.93 ± 1.388	Wrist curls + dynamometer contractions w/BFR bands	4	3	Active control	Grip strength	BFRT ↑ > TRAD
		TRAD—Traditional Strengthening	40	87	22.63 ± 1.608	Same exercises w/out BFR					
Wachholz et al., 2024 [37]	S	STG—Specific Training Group	10	0	28.7 ± 8.2	HIIT + app-based reaction/Stroop + anticipation (falling obj., videos) + grip (ball squeeze)	8	3	Active and passive control	Grip strength,	Grip ↑ in STG and GTG > NTG; no baseline–follow-up change overall
		GTG—General Training Group	9	0	27.4 ± 10.0	HIIT only (Tabata app, extra round vs. STG)					
		NTG—No Training Group	9	0	26.3 ± 7.4	No training					
Yoshitake et al. (2018) [43]	P	RPT—Random perturbation training	14	12	26.7 ± 4.9	Robot-assisted index finger posture; RPT = random perturbations	2	3	Active and passive control	Pinch strength, Force sense, Manual dexterity	RPT ↑ steadiness and dexterity; control → no change
		CFT—Constant force training	14			Neutral finger position vs. constant adduction force					
		Control	14			No training					

Legend: S = Strength training; P = Proprioceptive training; C = Combined training. ↑ = improvement; ↓ decrease; → = no change. NDH = Non-dominant hand; MVC = Maximum Voluntary Contraction; JPS = Joint Position Sense; BBT = Box and Block Test; NHPT = Nine-Hole Peg Test; PPB = Purdue Pegboard Test; JTHFT = Jebsen–Taylor Hand Function Test; MMDT = Minnesota Manual Dexterity Test; FTT = Finger Tapping Test; PNF = Proprioceptive Neuromuscular Facilitation; MP = Mental Practice; HVTF = Haptic + Vibrotactile Feedback; HF = Haptic Feedback; FDI = First Dorsal Interosseus; Bi-RBT = Bimanual Resistance Band Training; FES = Functional Electrical Stimulation; MI = Motor Imagery; AO = Action Observation; BM = Bimanual digit training; RH = Right-hand unimanual training; UB = Upper body; BFRT = Blood Flow Restriction Training; TRAD = Traditional Strength Training; RPT = Random Perturbation Training; STG = Specific Training Group; GTG = General Training Group; NTG = No Training Group.

### 3.2. Risk of Bias Assessment

**Figure 2:**
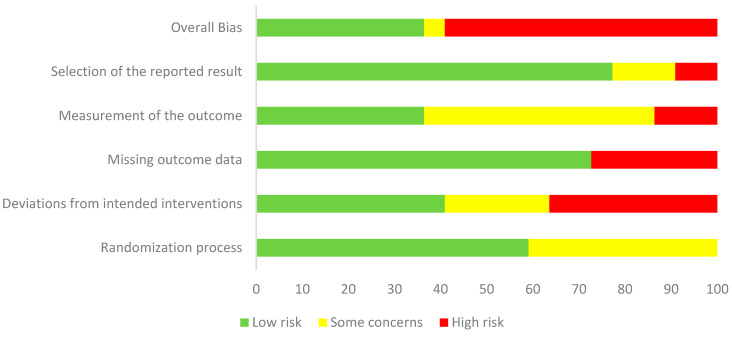
Weighted summary plot of risk of bias for the 22 included randomized controlled trials, assessed using the Cochrane Risk of Bias 2 tool.

Risk of bias was assessed for all 22 included randomized controlled trials using the Cochrane RoB 2 tool. Overall, 14 studies (64%) were judged to be at high risk of bias, 3 studies (14%) with some concerns, and 5 studies (23%) at low risk (Figure 2). The most frequent problems were related to incomplete reporting of the randomization process, deviations from intended interventions, and selective reporting.

**Figure 3:**
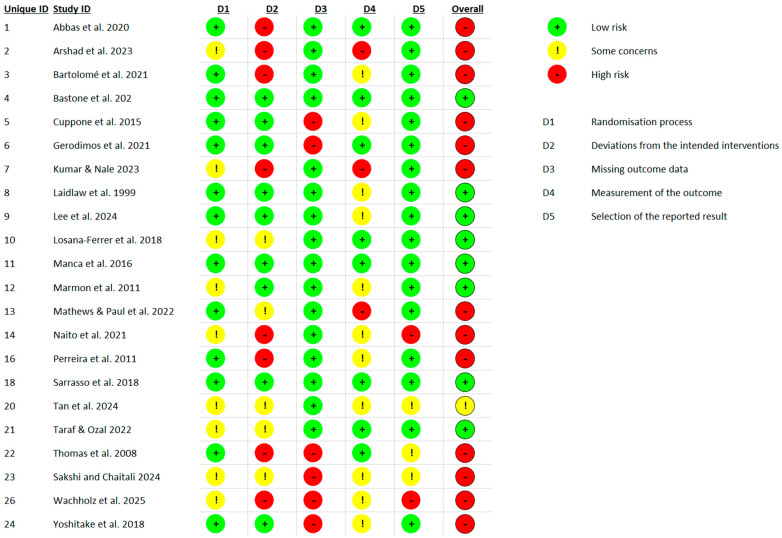
Traffic light plot showing risk of bias judgments for each domain in each of the 22 included RCTs, assessed using the Cochrane RoB 2 tool [1,6,7,8,26,27,28,29,30,31,32,33,34,35,36,37,38,39,40,41,42,43].

For the randomization process, 13 studies (59%) were rated as low risk, while 9 (41%) raised some concerns, most often due to insufficient detail on sequence generation or lack of allocation concealment. In deviations from intended interventions, 9 studies (41%) were at low risk, 5 (23%) had some concerns, and 8 (36%) were at high risk, with problems mainly arising from lack of blinding of participants and providers or unbalanced deviations from protocol. In the missing outcome data domain, 15 studies (68%) were judged low risk, 2 (9%) had some concerns, and 5 (23%) were at high risk, typically due to attrition without sufficient justification or inadequate handling of missing data. For measurement of the outcome, 20 studies (91%) were at low risk, 1 (5%) had some concerns, and 1 (5%) was at high risk, most often because of unblinded outcome assessors or potential influence of group knowledge on measurements. For selection of the reported result, 17 studies (77%) were judged low risk, 3 (14%) had some concerns, and 2 (9%) were high risk, usually due to the absence of trial registration or a pre-specified analysis plan. Taken together, these findings indicate that most trials were judged as having either “some concerns” or “high risk” of bias, particularly in the randomization process and deviations from intended interventions, which reduces the overall certainty of the pooled estimates (Figure 3).

### 3.3. Characteristics of Interventions

Training protocols were categorized as strength, proprioceptive, or combined based on their primary aims and methods.

Strength-based interventions constituted the largest group of protocols and exhibited considerable methodological diversity. Most programs targeted grip and forearm muscles using resistance-based modalities such as hand grippers, elastic bands, dumbbells, or bodyweight tasks (e.g., push-ups), aiming to improve force production, endurance, or muscle control. Training intensities were typically based on individual maximal capacities, such as percentages of one-repetition maximum (1-RM) or maximum voluntary contraction (MVC), and commonly progressed in volume or resistance over time (e.g., [1,6,27]). While many studies employed isometric or isotonic handgrip exercises in unilateral or bilateral formats (e.g., [7,8], others implemented multi-joint resistance protocols encompassing the entire upper limb [27,30,32,36], or used more localized interventions such as targeted first dorsal interosseus muscle training [40]. Some interventions also combined strength training with environmental manipulations like heat exposure [28], blood flow restriction [34], or visual/auditory feedback to refine motor control [41]. A smaller subset integrated functionally relevant tasks (e.g., carrying, twisting, or manipulating objects) with conventional resistance strategies to enhance strength in real-world contexts [30]. Across studies, protocols ranged from short-term (2–4 weeks) high-intensity regimens to longer, progressively loaded training plans lasting several months [27], underscoring the adaptability of strength training paradigms to various populations and research objectives.

Proprioceptive training targeted sensorimotor acuity and limb position sense using diverse approaches. Robotic systems provided precise force or haptic feedback (e.g., torque guidance or perturbations) to stimulate proprioceptive pathways in controlled conditions. Other protocols emphasized active control and sensory discrimination through rhythmic stabilization, complex finger coordination, or task-oriented movements. In older adults, interventions also aimed to modulate cortical inhibition mechanisms through bimanual digit training or functional exercises designed to promote sensory reweighting. While the specific modalities varied—from vibration and perturbation to isometric holds and FES-assisted movement—all shared the goal of enhancing fine motor control through augmented or focused proprioceptive input. In this category, Ghram et al. [44] applied proprioceptive neuromuscular facilitation (PNF) with or without daily mental practice, demonstrating that coupling sensorimotor drills with cognitive rehearsal can further enhance grip strength and muscle activation.

Combined training protocols integrated both strength-based and sensory-motor elements, aiming to simultaneously enhance neuromuscular activation and functional coordination. Several studies paired traditional resistance exercises with additional proprioceptive or functional tasks. For instance, Abbas et al. [1] introduced segmental vibration alongside isometric handgrip training, while Lee et al. [32] incorporated functional electrical stimulation (FES) during resistance band movements to target sensorimotor pathways. Others emphasized task-specific motor engagement: Mathews & Paul [30] employed functional activities such as object manipulation or buttoning to promote both muscular activation and fine motor control. Similarly, Losana-Ferrer et al. [35] combined motor imagery or action observation with isometric hand exercises, highlighting the role of cognitive-motor coupling. Newer multimodal approaches also emerged, such as the study by Wachholz et al. [37], where high-intensity interval training (HIIT) was combined with app-based reaction, Stroop, and anticipation tasks together with grip exercises, aiming to integrate cardiovascular, cognitive, and neuromuscular benefits.

### 3.4. Reported Outcomes and Availability for Meta-Analysis

Across the 22 included randomized controlled trials, outcome measures primarily focused on upper limb muscle function and sensorimotor performance. The most frequently reported variable was grip strength, assessed in 16 studies using hand dynamometry. Grip and pinch strength were assessed using a variety of handheld dynamometers and force transducers. The Jamar^®^ dynamometer was the most commonly used device (e.g., [27,33]), with most studies reporting outcomes in kilograms. Some studies [29,40,43] used strain gauge or piezoelectric transducers, with force reported in Newtons.

Pinch strength was explicitly measured in [33,41] typically using standardized pinch gauges. Despite variation in tools and units, strength outcomes were comparable and suitable for meta-analysis.

Manual dexterity was assessed in several studies using standardized tests evaluating both fine and gross motor skills. The Nine-Hole Peg Test (NHPT) was employed in [30,42] to assess fine finger movements, while [33,43] used the Purdue Pegboard Test (PPT) to evaluate coordinated hand and finger actions. The Jebsen-Taylor Hand Function Test (JTHFT), which simulates daily tasks, was administered in [38]. Ref. [30] also included the Box and Block Test (BBT) to assess gross manual dexterity. Additional tools included the Grooved Pegboard Test [29] and the Minnesota Manual Dexterity Test (MMDT) and Finger Tapping Test (FTT) [42].

Other outcomes, such as maximum voluntary contraction (MVC), joint position sense (JPS), force sense, or sensorimotor cortical excitability, were reported less consistently and with substantial variability in measurement techniques. These outcomes were reviewed qualitatively but excluded from the meta-analysis due to a lack of comparable data across studies or incomplete reporting of pre- and post-intervention statistics.

### 3.5. Performance Reported Through the Meta-Analysis

#### 3.5.1. Grip Strength

**Figure 4:**
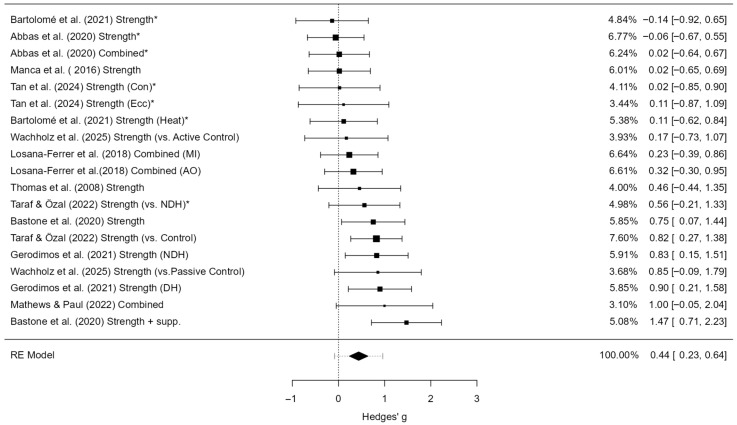
Forest plot of effect sizes (Hedges’ g) and 95% confidence and prediction intervals showing overall effect of hand-focused interventions on grip strength (random-effects model). Legend: 95% Prediction Interval [0.21 to 0.99]; SMD = Standardized Mean Difference; CI = Confidence Interval; PI = Prediction Interval; S = Strength training; P = Proprioceptive training; C = Combined training; NDH = Non-dominant hand (used as comparator in some trials); MVC = Maximum Voluntary Contraction; JPS = Joint Position Sense; BBT = Box and Block Test; NHPT = Nine-Hole Peg Test; PPB = Purdue Pegboard Test; JTHFT = Jebsen–Taylor Hand Function Test; MMDT = Minnesota Manual Dexterity Test; FTT = Finger Tapping Test. * Studies that used the non-dominant hand as a control group were adjusted by halving the sample size (N) in accordance with Cochrane guidance [1,6,7,26,27,28,30,35,36,37,41].

A total of 19 studies were included in the analysis. The observed standardized mean differences (SMDs) ranged from –0.14 to 1.47, with the majority of effects being positive (89%). The pooled effect size based on the random-effects model was statistically significant (g = 0.44, 95% CI [0.23, 0.64], z = 4.23, *p* < 0.0001), indicating a small to moderate positive effect of hand-focused interventions on grip strength. No significant heterogeneity was detected (Q(18) = 25.36, *p* = 0.115; τ^2^ = 0.06; I^2^ = 30.3%), suggesting that variability across studies was modest. Trim-and-fill analysis did not impute any additional studies, and the adjusted pooled effect remained identical to the observed effect. The 95% prediction interval ranged from –0.09 to 0.96, indicating that although the overall effect was positive, some future studies may still yield null or negative results (Figure 4).

An examination of studentized residuals revealed no outliers (threshold ± 3.01), and Cook’s distance values did not identify any overly influential studies. Furthermore, tests for funnel plot asymmetry were non-significant (rank correlation *p* = 0.679; regression test *p* = 0.797), providing no evidence of small-study effects or publication bias.

A sensitivity analysis was performed by re-estimating the models without correcting for unit-of-analysis dependency (i.e., treating trained and contralateral hands as independent). In this version, the pooled effect was slightly smaller (g = 0.39, 95% CI [0.19, 0.59]), heterogeneity was higher (I^2^ = 44.4%), and the Q-test indicated significant variability between studies (*p* = 0.021). The 95% prediction interval also widened (–0.21 to 0.99). Importantly, tests for funnel plot asymmetry suggested possible small-study effects in the uncorrected model (Egger *p* = 0.011; rank correlation *p* = 0.016), whereas this signal disappeared after applying the N/2 correction (Supplementary Table S1). These results indicate that the correction improved model stability and that the overall conclusions are robust to this source of potential bias.

For clinical interpretability, this standardized effect (g = 0.44) corresponds to an approximate mean improvement of around 4 kg in grip strength, based on a representative SD of 9.5 kg from Jamar dynamometer studies.

Sensitivity analyses using alternate pre–post correlations for change scores (r = 0.3 and r = 0.7) yielded comparable pooled effects (g = 0.38 to 0.55) with stable significance; details are provided in Supplementary Table S2.

**Subgroup estimate.** Moderator analyses revealed that age, training type, and comparator type influenced the magnitude of the intervention effect (Table 2). Older adults showed significantly greater effects (g = 0.97, *p* < 0.001) compared to younger adults, who demonstrated a smaller but still significant effect (g = 0.26, *p* = 0.009). Strength training alone produced a moderate and statistically significant effect (g = 0.47, *p* < 0.001), while the combination of strength and proprioceptive training yielded a smaller, non-significant effect (g = 0.28, *p* = 0.107). The type of comparator also moderated the results: studies using passive control groups reported large, significant effects (g = 0.63, *p* < 0.001), whereas comparisons to the untrained hand showed negligible and non-significant effects (g = 0.08, *p* = 0.595). These findings suggest that training effects appeared more pronounced in older adults, particularly when strength-based protocols were applied and when outcomes were compared against passive rather than internal controls. However, several of these subgroups included fewer than ten studies, and the corresponding prediction intervals were wide, indicating substantial uncertainty. Consequently, the moderator effects should be considered exploratory and interpreted with caution.

**Meta-regression.** With age group as a moderator (k = 19) indicated that older adults experienced larger effects compared to younger adults (β = 0.71, SE = 0.20, z = 3.63, *p* < 0.001). The estimated effect was small and non-significant in younger adults (g = –0.46, 95% CI [–0.97, 0.05]) but positive and significant in older adults (g = 0.26, 95% CI [0.09, 0.43]). Including age as a moderator reduced residual heterogeneity to zero (I^2^ = 0%).

Meta-regression with training type as moderator (k = 19) indicated no significant difference between strength-based and combined protocols (β = –0.17, SE = 0.25, *p* = 0.49). The estimated effect was moderate and statistically significant for strength training (g = 0.65, 95% CI [0.02, 1.28]), while the effect for combined training was smaller and did not reach significance (g = 0.48, 95% CI [–0.02, 0.98]). Residual heterogeneity remained moderate (τ^2^ = 0.06, I^2^ = 30.7%). These findings are consistent with the subgroup analyses, which showed a significant effect for strength training (g = 0.47) and a smaller, non-significant effect for combined training (g = 0.28).

Meta-regression with comparator type as moderator (k = 19) confirmed a significant moderating effect (β = –0.55, SE = 0.18, *p* = 0.002). Subgroup analyses showed large and statistically significant effects when interventions were compared against passive controls (g = 0.63, 95% CI [0.29, 0.97]) but negligible and non-significant effects when compared against the untrained hand (g = 0.08, 95% CI [–0.22, 0.38]). Including comparator type in the model reduced heterogeneity to zero (τ^2^ = 0, I^2^ = 0%), indicating that comparator type fully explained the between-study variability.

Overall, the moderator analyses indicate that intervention effects were larger in older adults than younger adults, most robust for strength-based protocols, and more detectable when compared with passive rather than internal controls. However, given the limited number of studies per subgroup and potential confounding between moderators, these findings should be considered exploratory and interpreted with caution.

Full regression coefficients (β, SE, and *p*-values) for age group, training type, and comparator type are reported in Supplementary Table S3.

#### 3.5.2. Pinch Strength

**Figure 5:**
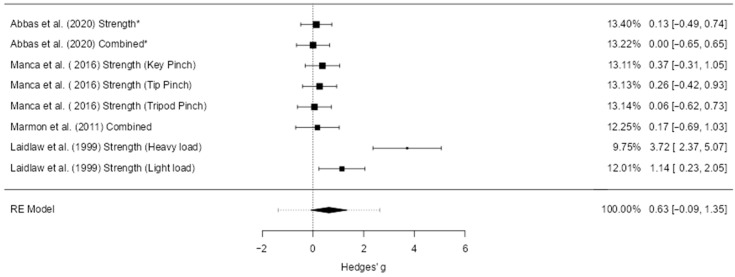
Forest plot of effect sizes (Hedges’ g) and 95% confidence and prediction intervals showing overall effect of hand-focused interventions on pinch strength (random-effects model). Legend: 95% Prediction Interval [–1.38 to 2.63]; SMD = Standardized Mean Difference; CI = Confidence Interval; PI = Prediction Interval; S = Strength training; P = Proprioceptive training; C = Combined training; NDH = Non-dominant hand (used as comparator in some trials); MVC = Maximum Voluntary Contraction; JPS = Joint Position Sense; BBT = Box and Block Test; NHPT = Nine-Hole Peg Test; PPB = Purdue Pegboard Test; JTHFT = Jebsen–Taylor Hand Function Test; MMDT = Minnesota Manual Dexterity Test; FTT = Finger Tapping Test. * Studies that used the non-dominant hand as a control group were adjusted by halving the sample size (N) in accordance with Cochrane guidance [1,29,40,41].

A total of eight studies (k = 8) were included in this analysis. The observed standardized mean differences ranged from 0.00 to 3.72, with the majority of estimates being positive (88%). The pooled effect was positive but not statistically significant (g = 0.63, 95% CI [–0.09, 1.35], z = 1.71, *p* = 0.088), indicating a trend toward a favorable effect that did not reach significance. Substantial heterogeneity was detected (Q(7) = 29.32, *p* < 0.001; τ^2^ = 0.91; I^2^ = 86.3%), and the 95% prediction interval ranged from –1.38 to 2.63, suggesting wide variation in possible true effects across studies (Figure 5). One study ([40]; Strength, heavy load) exceeded the studentized residual threshold (±2.73) and was flagged as a potential outlier; the same study was also identified as overly influential based on Cook’s distance.

Publication bias analyses suggested potential small-study effects. The fail-safe N was 34, indicating that 34 unpublished null studies would be needed to nullify the observed trend. The rank correlation test (*p* = 0.014) and Egger’s regression (*p* < 0.001) both supported funnel plot asymmetry. However, trim-and-fill analysis did not impute any additional studies, and the pooled effect estimate was unchanged. This suggests that while small-study effects may have influenced the distribution of study results, they did not materially alter the pooled effect.

#### 3.5.3. Manual Dexterity

**Figure 6:**
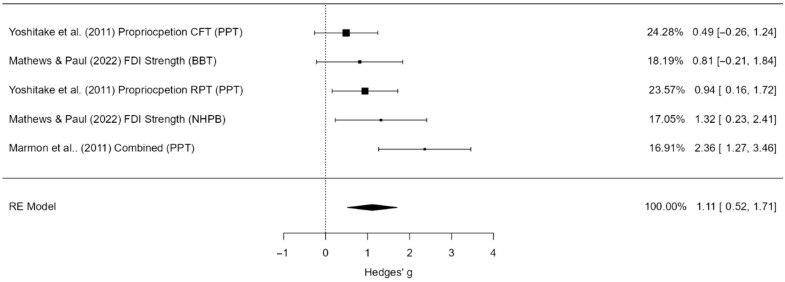
Forest plot of effect sizes (Hedges’ g) and 95% confidence and prediction intervals for the effect of hand-focused strength and proprioceptive training on manual dexterity. Legend: 95% Prediction Interval [–0.01 to 2.23]; SMD = Standardized Mean Difference; CI = Confidence Interval; PI = Prediction Interval; S = Strength training; P = Proprioceptive training; C = Combined training; NDH = Non-dominant hand (used as comparator in some trials); MVC = Maximum Voluntary Contraction; JPS = Joint Position Sense; BBT = Box and Block Test; NHPT = Nine-Hole Peg Test; PPB = Purdue Pegboard Test; JTHFT = Jebsen–Taylor Hand Function Test; MMDT = Minnesota Manual Dexterity Test; FTT = Finger Tapping Test [29,30,43].

A total of five studies (k = 5) were included in this analysis. The pooled standardized mean difference was large and statistically significant (g = 1.11, 95% CI [0.52, 1.71], *p* < 0.001), indicating a strong effect of the intervention (Figure 6). Moderate heterogeneity was observed (I^2^ = 51.2%), though the Q-test was not statistically significant. The 95% prediction interval ranged from –0.01 to 2.23, suggesting that effects may vary across settings. No outliers or overly influential studies were identified. Funnel plot analysis suggested possible small-study effects or publication bias, as indicated by the regression test (*p* = 0.047), although the rank correlation test did not reach significance (*p* = 0.083).

Publication bias analyses provided mixed results. The fail-safe N was 47, indicating that 47 unpublished null studies would be required to eliminate the observed effect. Egger’s regression suggested possible asymmetry (*p* = 0.047), whereas the rank correlation test did not reach significance (*p* = 0.083). Trim-and-fill analysis did not impute any additional studies, and the pooled effect size remained unchanged. Thus, although small-study effects cannot be excluded, the overall large effect estimate for manual dexterity appears robust.

## 4. Discussion

This systematic review and meta-analysis evaluated the effects of hand-focused strength and proprioceptive training on upper limb function in healthy younger and older adults. Across 22 randomized controlled trials, the findings indicate small-to-moderate improvements in grip strength (g = 0.44, ≈+4 kg) and large gains in manual dexterity (g = 1.11). These magnitudes suggest that relatively brief, task-specific interventions yielded meaningful benefits, particularly in older adults who improved more than younger adults Strength-only protocols produced consistent, statistically significant effects, whereas combined programs showed smaller, non-significant effects; however, meta-regression did not confirm a significant difference between training types. By contrast, the age effect was robust, with meta-regression confirming that older adults benefitted significantly more than younger adults. Comparator type also remained a significant moderator in meta-regression, with larger effects for passive controls than for untrained hands. Although pinch strength showed a positive trend, high heterogeneity and small-study effects limited confidence in this result. Together, these findings provide evidence that targeted hand training can improve both strength and fine motor skills in healthy populations, with especially strong potential for preserving function in aging.

The greater responsiveness of older adults to hand-focused strength training likely reflects a combination of lower baseline strength and dexterity, and a higher potential for neuromuscular adaptation. Age-related declines in grip strength, dexterity, and sensorimotor function—accelerating particularly after the age of 65—create a larger margin for improvement [8,20,27]. Older adults retain a substantial capacity for neuromuscular plasticity, demonstrating comparable increases in protein synthesis rates and neural adaptations to resistance training as younger individuals [27,45]. Short-term, high-intensity protocols can help overcome the greater “neural deficit” seen in this group, enabling fuller activation of muscle potential and reversal of some motor function decline [9,46]. These age-related mechanisms align with our statistical findings, as the moderator analysis confirmed that age significantly moderated training effects.

Beyond age, our analyses also indicated that the type of training modality may influence outcomes, although evidence was mixed. Strength-only protocols showed significant effects in subgroup analyses, whereas combined protocols did not, but meta-regression did not confirm a statistically significant difference between the two. Strength training may improve the mechanical efficiency of grip and pinch actions through multiple pathways [2,41]. Early gains are often driven by neural factors such as increased motor unit recruitment, higher maximal discharge rates, and improved coordination of intrinsic and extrinsic hand muscles, with structural hypertrophy contributing over longer durations [30]. Enhanced neuromuscular coordination allows more efficient activation patterns during functional and sport-specific hand tasks, while bimanual training can facilitate control improvements in both hands [30,32].

Proprioceptive training targets the sensory component of hand function by refining afferent input from muscles, tendons, and joint receptors [2,42,43]. Such training—using methods like joint position matching, perturbation-based grip tasks, and vibrotactile feedback—can improve force steadiness, positional acuity, and fine finger coordination [43,47]. These changes are linked to reduced force variability, reactivation of cortical areas involved in motor control, and increased neural efficiency, all of which are closely associated with improvements in manual dexterity [19,31].

The lower effect sizes observed for combined strength–proprioception interventions may reflect a trade-off in stimulus specificity. Adding proprioceptive elements to a strength regimen can reduce the overall training volume dedicated to strength development, potentially attenuating grip strength gains. Moreover, when sensory input is poorly timed or excessive, it may interfere with motor learning, as evidenced by findings where enhanced cutaneous stimulation hindered interlimb strength transfer. Some strength-focused protocols have also demonstrated trade-offs, such as increasing force capacity at the expense of finger independence [23,46], indicating that training outcomes are highly dependent on the targeted adaptation [7].

The negligible between-hand differences observed when comparing the trained to the untrained limb in our meta-analysis may indicate some degree of cross-education, whereby unilateral training elicits gains in the contralateral, non-trained hand. Such transfer could partially explain why effect sizes were markedly larger in comparisons with passive controls. Even modest improvements in the untrained hand would reduce the between-hand contrast, making this comparator less sensitive for detecting training-induced gains [6,28,41]. Meta-regression confirmed that comparator type significantly moderated effect sizes, reinforcing that passive control groups are more sensitive for detecting training-induced improvements than internal untrained-hand comparators.

Our findings align, in several respects, with prior reviews and further extend them. For grip strength, our pooled effect size (g = 0.44) is broadly consistent with the small intervention effects reported in healthy older adults [10] (SMD = 0.28, cited in Abe et al., 2023) [17], although we observed substantially larger gains in older (g = 0.97) than younger adults (g = 0.26). This age-related contrast supports Abe et al.’s hypothesis [17] that resistance training has negligible impact in younger adults. For manual dexterity, our large pooled effect (g = 1.11) exceeds the modest SMDs for fine motor dexterity reported in Parkinson’s disease populations [13] and is consistent with the substantial percentage improvements in proprioceptive and sensorimotor outcomes described by Aman et al. [12]. In contrast, Magni, McNair, and Rice [48] found no functional gains in hand osteoarthritis, likely reflecting disease-specific limitations.

Strength-only protocols elicited greater grip strength gains (g = 0.42) than combined strength–proprioception programs (g = 0.23, non-significant), whereas Aman et al. [12] suggested that multimodal training might best enhance overall motor function. This divergence may reflect outcome specificity, and our meta-regression did not detect a statistically significant difference between the two training types. Finally, our subgroup analysis by comparator type revealed markedly larger effects against passive controls (g = 0.65) than against the untrained hand (g = 0.08), the latter likely influenced by cross-education effects reducing between-hand contrasts—an aspect seldom addressed in previous reviews. The significance of comparator type as a moderator was also confirmed in the meta-regression.

This review also revealed variation in whether interventions primarily targeted intrinsic or extrinsic muscles of the hand. Several studies isolated the FDI to represent intrinsic muscle training, showing benefits for pinch strength and steadiness [40,41,49]. By contrast, most protocols emphasized extrinsic wrist and forearm musculature through wrist curls, handgrip exercises, or resisted flexion–extension tasks, improving overall grip strength [6,26,34,35]. Other programs combined finger and grip movements (e.g., [7,8,33], engaging both groups but without an explicit focus on one or the other. Importantly, none of the studies directly compared intrinsic- versus extrinsic-focused regimens, which represents an opportunity for future research to clarify whether fine-motor intrinsic training confers distinct benefits beyond global strengthening of extrinsic muscles.

Several factors should be considered when interpreting these findings. Methodological quality was generally low, with 46% of trials rated at high risk of bias, 46% with some concerns, and only 9% at low risk, mainly due to lack of blinding and incomplete reporting of allocation concealment. Measurement heterogeneity—arising from the use of different dynamometers, pinch gauges, and dexterity tests—may have further inflated between-study variance. Although the pooled effect for manual dexterity was large and statistically significant, confidence in this estimate is limited by the small number of contributing trials (k = 5) and indications of small-study effects. For pinch strength, the non-significant pooled effect (k = 8) was further undermined by high heterogeneity and evidence of publication bias. In addition, insufficient and inconsistent reporting of MVC, JPS, and force sense precluded quantitative synthesis for these outcomes, underscoring the need for standardized measurement protocols. Another important limitation is the absence of prospective registration in PROSPERO or OSF, which may reduce transparency and should be considered when interpreting the findings. While restricting inclusion to RCTs improved internal validity, it reduced the breadth of the available evidence. Finally, heterogeneity in intervention duration, intensity, and frequency may limit the generalizability of the findings, and the small number of studies in some subgroups lowers the power to detect moderator effects.

While a formal GRADE evaluation was not performed, the overall certainty of evidence in this review is likely to be low. This is primarily due to the high or unclear risk of bias in many included studies, the small number of trials for certain outcomes, and imprecision reflected in wide confidence and prediction intervals. These limitations reduce confidence in the pooled estimates and highlight the need for larger, well-designed RCTs to strengthen the evidence base.

## 5. Conclusions

This systematic review and meta-analysis indicates that hand-focused strength and proprioceptive training can improve grip strength and manual dexterity in healthy adults, with effects most pronounced in older participants and in trials using strength-based protocols. However, the evidence for pinch strength was inconsistent, and comparator choice strongly influenced outcomes. These findings highlight the potential of targeted hand training for preserving and enhancing upper limb function, particularly in aging. At the same time, the overall certainty of evidence is low, as most included trials were at high risk of bias, sample sizes were small, and outcome measures were heterogeneous. Future well-designed randomized controlled trials with standardized protocols are needed to confirm these preliminary findings and strengthen the evidence base.

### Future Directions

Future work should focus on high-quality, adequately powered RCTs with clear randomization, allocation concealment, and blinding where feasible. Direct head-to-head comparisons of strength, proprioceptive, and combined protocols in the same population are needed to clarify modality-specific effects. Standardized, validated outcome measures for grip strength, pinch strength, dexterity, MVC, JPS, and force sense should be adopted to facilitate comparison across studies. Exploration of dose–response relationships and long-term retention of training effects would further inform evidence-based recommendations for sport performance optimization and healthy aging.

## Figures and Tables

**Table 2:** Subgroup analysis of effect sizes (Hedges’ g) by age, training type, and comparator for grip strength.

Moderator	Subgroup	k	g (95% CI)	*p*	I^2^
Age					
	Younger adults	14	0.26 (0.06, 0.45)	**0.009**	0%
	Older adults	5	0.97 (0.64, 1.3)	**<0.001**	0%
Training type					
	Strength	15	0.47 (0.23, 0.72)	**<0.001**	37.45%
	Combined (strength + proprioception)	4	0.28 (−0.06, 0.63)	0.107	0%
Comparator type					
	Passive control	12	0.63 (0.40, 0.87)	**<0.001**	21.77%
	Untrained hand (internal)	7	0.08 (−0.21, 0.36)	0.595	0%

Legend: k = number of study arms; I^2^ = heterogeneity. Bolded values indicate statistically significant results.

## Data Availability

Template data collection forms, extracted data from included studies, datasets used in all analyses can be provided by the corresponding author upon reasonable request.

## Supplementary Materials

The following supporting information can be downloaded at https://www.mdpi.com/article/10.3390/jcm14196882/s1: Table S1. Sensitivity analysis comparing pooled estimates before and after bilateral correction (Cochrane N/2 adjustment). Table S2. Pooled estimates shown for r = 0.3, 0.5 (main), and 0.7. Table S3. Meta-regression results for moderators of intervention effects on grip strength (k = 19 study arms).
