# Supplementary material for: Hand-Focused Strength and Proprioceptive Training for Improving Grip Strength and Manual Dexterity in Healthy Adults: A Systematic Review and Meta-Analysis
Source: J Clin Med. 2025 Sep 28;14(19):6882. doi: 10.3390/jcm14196882 (PMC12524766; doi:10.3390/jcm14196882)
Supplement: Supplementary file 1 [file jcm-14-06882-s001.zip › jcm-3849834-supplementary.pdf]

Table S1. Sensitivity analysis comparing pooled estimates before and after bilateral correction (Cochrane N/2 adjustment).

	Before bilateral correction	After bilateral correction
k	19	19
Hedges' g	0.39	0.44
95% CI	0.19 – 0.59	0.23 – 0.64
I^2	44.43%	30.30%
τ^2	0.083	0.06
Egger *p*	0.011	0.797

Table S2. Pooled estimates shown for r = 0.3, 0.5 (main), and 0.7.

Assumed r	k	Hedges' g	95% CI	p	τ^2	I^2 (%)	95% Prediction Interval	Egger p
0.3	19	0.376	0.2012 to 0.5508	<0.0001	0.0134	8.9	0.0900 to 0.6621	0.947
0.5	19	0.437	0.2346 to 0.6400	<0.0001	0.0604	30.3	–0.0853 to 0.9599	0.796
0.7	19	0.551	0.2960 to 0.8059	<0.0001	0.1708	54.5	–0.2984 to 1.4002	0.52

Table S3. Meta-regression results for moderators of intervention effects on grip strength (k = 19 study arms).

Moderator	β Estimate	SE	Z	p	95% CI Lower	95% CI Upper
Age group	0.71	0.20	3.63	<0.001	0.32	1.10
Training type	–0.17	0.25	–0.69	0.49	–0.66	0.32
Comparator type	–0.55	0.18	–3.09	0.002	–0.90	–0.20
